# Sucrose targets clathrin-mediated endocytosis kinetics supporting cell elongation in *Arabidopsis thaliana*

**DOI:** 10.3389/fpls.2022.987191

**Published:** 2022-10-18

**Authors:** Claudio Osorio-Navarro, Jorge Toledo, Lorena Norambuena

**Affiliations:** ^1^Department of Biology, Facultad de Ciencias, Plant Molecular Biology Centre, Universidad de Chile, Santiago, Chile; ^2^Red de Equipamiento Científico Avanzado (REDECA), Faculty of Medicine, Universidad de Chile, Santiago, Chile

**Keywords:** sucrose, cell elongation, clathrin-mediated endocytosis (CME), trafficking, clathrin

## Abstract

Sucrose is a central regulator of plant growth and development, coordinating cell division and cell elongation according to the energy status of plants. Sucrose is known to stimulate bulk endocytosis in cultured cells; however, its physiological role has not been described to date. Our work shows that sucrose supplementation induces root cell elongation and endocytosis. Sucrose targets clathrin-mediated endocytosis (CME) in epidermal cells. Its presence decreases the abundance of both the clathrin coating complex and phosphatidylinositol 4,5-biphosphate at the plasma membrane, while increasing clathrin complex abundance in intracellular spaces. Sucrose decreases the plasma membrane residence time of the clathrin complex, indicating that it controls the kinetics of endocytic vesicle formation and internalization. CME regulation by sucrose is inducible and reversible; this on/off mechanism reveals an endocytosis-mediated mechanism for sensing plant energy status and signaling root elongation. The sucrose monosaccharide fructose also induces CME, while glucose and mannitol have no effect, demonstrating the specificity of the process. Overall, our data show that sucrose can mediate CME, which demonstrates that sucrose signaling for plant growth and development is dependent on endomembrane trafficking.

## Introduction

Plants obtain their carbon skeletons and energy from sugars synthesized through photosynthesis. Nutrient balance and hormone signaling are closely integrated with sugar levels, regulating plant growth and development (Sakr et al., 2018). Sugars participate in different processes at the transcriptional and post-transcriptional levels, with glucose perception and signaling being the best-documented case in eukaryotes (Wingler, 2018). In many plants, sucrose is the most abundant sugar mobilized from photosynthetically active tissues to sink organs (Durand et al., 2018). Sucrose acts as a signaling molecule that regulates cellular processes with impacts at the physiological level (Chiou and Bush, 1998; Loreti et al., 2000; Kircher and Schopfer, 2012). For example, sucrose plays a role in protein accumulation, anthocyanin biosynthesis, flowering induction, and root growth (Yoon et al., 2021). Sucrose-mediated transcriptional regulation has been extensively studied, and the roles of WRKY, MYB, and bZIP-type transcription factors in specific responses have been described (Teng et al., 2005; Kang et al., 2010; Nagata et al., 2012). In contrast, the signaling mechanism of sucrose at the cellular level is largely unknown.

Long-distance sucrose mobilization depends on plasma-membrane-resident sucrose transporters that assist the loading and unloading of sucrose from the phloem (Durand et al., 2018). SUCROSE TRANSPORTERS (SUTs) and SUCROSE CARRIERS (SUCs) are H^+^/sucrose symporters involved in loading sucrose into the phloem as well as its uptake in sink organs (Ayre, 2011); SUC1 and SUT4 are implicated in specific sucrose signaling (Li et al., 2012; Shin et al., 2013). Interestingly, SUTs are internalized from the plasma membrane into endosomal compartments depending on redox conditions (Krügel et al., 2008; Liesche et al., 2008; Garg et al., 2020). Also, G-protein signaling associated with both sucrose and glucose sensing is activated only after REGULATOR OF G-PROTEIN SIGNALING 1 (RGS1) is endocytosed (Urano et al., 2012). This suggests that the presence of sucrose in the extracellular space could stimulate the endocytosis trafficking pathway. Indeed, sucrose induces fluid-phase endocytosis in a concentration-dependent manner in cultured *Acer pseudoplatanus* cells (Etxeberria et al., 2005).

In plants, two types of endocytosis have been described and are distinguished by their dependency on the vesicle-coating complex clathrin (Chen et al., 2011; Fan et al., 2015). Clathrin-mediated endocytosis (CME) is the more relevant pathway for the flux of components in mammalian cells (Bitsikas et al., 2014). Moreover, CME is the best-characterized pathway for the selection and internalization of plasma membrane cargo in plant cells. CME is initiated through cargo selection from the plasma membrane domains enriched in phosphatidylinositol 4,5-biphosphate (PI(4,5)P_2_) by cytoplasmic adaptor complexes. ADAPTOR PROTEIN COMPLEX-2 (AP2) recognizes endocytic cargo motifs in the cargo protein through its AP2μ2 subunit. The interaction and concentration of cargo proteins, AP2 and clathrin complexes, induces plasma membrane deformation, which generates a clathrin-coated vesicle that is then internalized (Fan et al., 2015). A myriad of biotic, abiotic, and endogenous stimuli modulate endocytosis in plant cells, allowing the integration and coordination of physiological responses (Chen et al., 2011).

Here, we show that exogenous sucrose induces cell elongation and endocytic trafficking in the epidermal cells of *Arabidopsis thaliana* (Arabidopsis) roots. In particular, sucrose targets CME by modulating the kinetics of vesicle formation and internalization. This effect is inducible and reversible, suggesting that CME plays a role in the physiological response to fluctuations in sucrose availability. The effect is specific to sucrose and its monosaccharide fructose, indicating that these two sugars are involved in a specific signaling mechanism supported by endocytic trafficking for cell elongation and consequently, root growth.

## Materials and methods

### Plant materials and growth conditions

*Arabidopsis thaliana* wild type (Col-0), the double mutant *clc2-1 clc3-1* (Wang et al., 2013) and transgenic lines *pCLC2:CLC2-GFP* (Wang et al., 2013), *pAP2*μ*2:AP2*μ*2-YFP* (Bashline et al., 2013), *pPIN2:PIN2-GFP* (Xu and Scheres, 2005), and *pUBQ10:mCIT-1xPH*^*PLC*δ1^ (Simon et al., 2014) were used in this study. Surface-sterilized seeds were stratified in the dark at 4°C for 48 h. Seeds were sown on solid medium (SM) containing 0.44% Murashige and Skoog salts with vitamins (PhytoTecnology Laboratories™), 0.05% MES (pH 5.7), 0.01% myo-inositol, and 0.7% phytoagar (PhytoTecnology Laboratories™). Liquid medium (LM) was prepared with the same composition as SM, but without phytoagar. Seedlings were grown vertically on SM in a growth chamber at 23°C with a 16-h light/8-h dark photoperiod.

### Primary root phenotypes

Seedlings growing on plates were scanned using an EPSON Perfection V600 Photo scanner. To evaluate cell size, the plasma membrane was labeled with a pulse of FM4-64 (4 μM; Invitrogen) and visualized by confocal microscopy. Root length and cell size were measured manually with the Fiji software (Schindelin et al., 2012).^1^ The cell elongation index was obtained as the ratio between the height and width of each cell.

### Endocytosis kinetics

Seedlings were treated with SM supplemented with 4 μM FM4-64 (Invitrogen) for 10 min at 4°C for plasma membrane labeling. Then, seedlings were washed with fresh LM for 5 min to remove excess FM4-64 (time zero). Seedlings were incubated at 22°C to allow plasma membrane internalization. Temporal FM4-64 internalization in root epidermal cells was evaluated by confocal microscopy. Images were taken after 5, 15, and 30 min of incubation.

### Confocal microscopy

Whole Arabidopsis seedlings were mounted in LM on a slide. Root cells were visualized with a confocal microscope (Zeiss LSM 710) with an EC Plan-Neofluar 40 × /1.30 oil DIC M27 objective lens. The parameters were laser 543 nm/emission>588–596 nm and laser 488 nm/emission 533–568 nm for FM4-64 and the fluorescent proteins GFP/YFP, respectively.

### Image processing and fluorescence signal quantification

Confocal images were pseudo-colored using the rainbow RGB LUT for informing the intensity of fluorescence markers. For signal quantification, the mean fluorescence values were obtained from manually defined regions of interest (ROIs) using Fiji (see footnote 1; Schindelin et al., 2012).

### Total internal reflection fluorescence microscopy

Whole Arabidopsis seedlings were mounted in LM on a slide. The cover slip was placed over the root tip. Epidermal cells at the root transition zone were imaged with a Zeiss AxioObserver.Z1 microscope (laser 483–493 nm/emission 500–550 nm) with an Alpha Plan-Apochromat 100 × /1.46 oil DIC (UV) M27 objective lens. The incident angle was set at 68°. Images were obtained at 100-ms intervals to generate 2–5-min movies. The lifetime was calculated from tracking analysis of endocytic events. Foci tracking was performed using the Trackmate plug-in for ImageJ. For spot detection, a Laplacian of Gaussian (LoG) filter was used with Blob diameter detector. The parameter “estimated blob diameter” was set to 0.8 μm and “sub-pixel localization” was activated. For tracking, generation of 2D trajectory tracks from particle locations was done using a simple linear assignment algorithm “LAP Tracker.” Gap closing was allowed with a maximum closing distance of 1 μm and a maximum frame gap of two frames. The maximum linking distance was set to 1 μm and statistical values was grouped using Gallery Plot options in ImarisVantage (Bitplane).

### Data and statistical analysis

All graphs show mean and standard error. Sample size and statistical methods are included in each figure legend. The statistical significance was calculated by analysis of variance and by Student’s *t*-test. All analyses were performed with GraphPad Prism 9.3.1 (GraphPad, San Diego, CA, USA).

## Results

### Sucrose promotes root cell elongation

**FIGURE 1:**
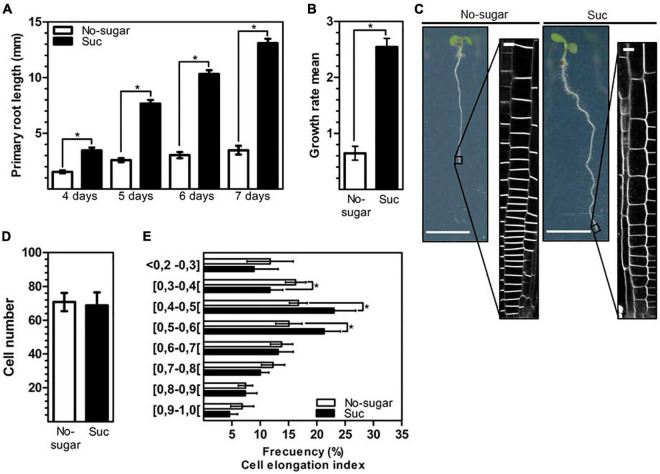
Elongation of root epidermal cells is promoted by sucrose. Wild-type Arabidopsis (Col-0) seeds were sown on solid medium without sugar (No-sugar, white bar) or supplemented with 60 mM sucrose (Suc, black bar) and grown for several days. **(A)** Primary root length of seedlings was measured in *n* ≥ 20 seedlings per replicate. **(B)** Growth rate per day of seedling roots from day 4 to day 7 in **(A)**. **(C)** Epidermal cells at the root tip were visualized by plasma membrane FM4-64-staining of seedlings after 7 days. Representative image of seedlings in each condition are shown. Bar: 5 mm. Representative confocal images are shown (*n* ≥ 20 seedlings under each condition). Bar, 10 μm. **(D,E)** Cell height and width were measured for epidermal cells located up to 300 μm above the quiescent center of the primary root of seedlings in **(B)**. The ratio of cell height/width was considered the cell elongation index. **(D)** The number of meristematic cells (ratio height/width ≤ 1) of the seedling primary root in **(B)** was scored. **(E)** The number of meristematic cells is shown as the percentage of cells that display a range of cell elongation index values. *n* ≥ 15 seedlings were scored in either condition. **(A,B,D,E)** Error bars represent standard error for three independent biological replicates. Student’s *t*-test; *p< 0.001.

The photosynthate sucrose is necessary and sufficient to promote the growth of Arabidopsis primary roots (Kircher and Schopfer, 2012). Organ growth requires a fine balance between cell division and expansion. Primary root growth is mainly due to the expansion of cells after reaching the transition root region. In our study, wild-type Arabidopsis seedlings developed longer roots when seeds germinated and grew in the presence of 60 mM sucrose than when no sugar was added (Figure 1A). The effect of sucrose on root growth was first observed just after germination (about day 2). At day 4 after sowing, the presence of sucrose in the medium resulted in primary roots that were 44.5% longer than those grown in the absence of sugar. The increase in root length with sucrose progressively increased to 55.5, 66.2, 70.6, and 73.4% at days 4, 5, 6, and 7, respectively (Figure 1A). Root length increased by 21.5% from day 4 to day 7 in seedlings grown in no-sugar medium, but by 62.1% in seedlings grown in sucrose-supplemented medium over the same period (Figure 1A); thus, a higher growth rate was observed in sucrose medium than in no-sugar medium (Figure 1B). The root growth promotion induced by 60 mM sucrose was the same magnitude when the treatment was performed with 30 or 90 mM of this sugar (Supplementary Figure 1). Microscopy revealed that sucrose-grown seedlings had a more extended meristem zone and longer cells than seedlings grown in no-sugar medium (Figure 1C). The increase in meristem zone size could not be explained by stimulation of cell division since the presence of sucrose had no effect on the number of meristematic cells (Figure 1D). Most likely, the increased meristem size was due to sucrose-induced cell elongation. Therefore, we evaluated the cell elongation index as the ratio between the height and width of each cell above the quiescent center up to cells with the same height and width as the criteria for the end of the meristematic zone. Seedlings grown in sucrose medium had a significantly greater number of cells with an elongation index of 0.4–0.6 than seedlings grown in no-sugar medium (Figure 1E). In contrast, shorter cells with an elongation index below 0.4 were more abundant in seedlings grown in no-sugar medium. Therefore, the effect of sucrose on the root growth rate was mainly due to the promotion of cell elongation.

### Plasma membrane internalization is induced by sucrose in root epidermal cells

**FIGURE 2:**
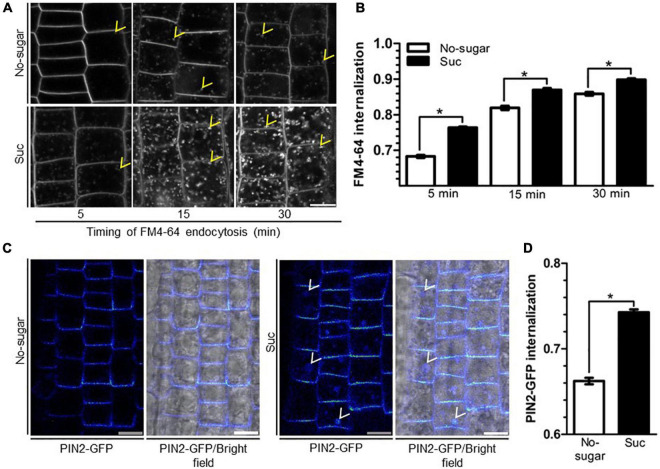
Sucrose induces plasma membrane endocytosis in root epidermal cells. Arabidopsis wild-type (Col-0) **(A,B)** or PIN2-GFP **(C,D)** seedlings were sown on sugar-free (No-sugar) or 60 mM sucrose-supplemented (Suc) solid medium and grown for 7 days. **(A)** Endocytosis kinetics of FM4-64 were evaluated by confocal imaging of epidermal cells after 5, 15, or 30 min of FM4-64 plasma membrane labeling. About 15 seedlings were evaluated for either condition in experimental triplicates. Representative images are shown. Yellow arrowheads indicate representative FM4-64-stained endosomes. Bar, 10 μm. **(B)** FM4-64 uptake was quantified by measuring intracellular and whole-cell FM4-64 signal in each imaged cell. Results are expressed as the ratio between both values. **(C)** Seedlings were incubated in 50 μM cycloheximide to exclusively visualize PIN2-GFP endocytic trafficking. After 30 min, seedlings were incubated in the dark for 6 h to induce PIN2 endocytosis toward the vacuole. PIN2-GFP cell distribution was evaluated by confocal microscopy. Representative confocal images from *n* = 15 seedlings in each condition performed in experimental triplicates are shown as images of Rainbow RGB LUT (Fiji). White arrowheads indicate PIN2-GFP-labeled vacuoles. Bar, 10 μm. **(D)** PIN2-GFP internalization was calculated as the intracellular signal relative to the whole-cell signal of seedlings in **(C)**. **(B,D)** Standard error for three independent biological replicates is shown. Two-tailed Student’s *t*-test; *p< 0.001.

Chemical or genetic disruption of trafficking pathways leads to multiple defects in growth and development (Norambuena and Tejos, 2017). Endocytosis is required during cell elongation and division, allowing the specific distribution of lipids and proteins (Dhonukshe et al., 2006; Zhang et al., 2019). Sucrose induces bulk endocytosis in cultured plant cells, suggesting that this stimulus could be associated with nutrient uptake from the extracellular space (Etxeberria et al., 2005). Therefore, we analyzed whether sucrose induces endocytosis in root cells by analyzing the internalization kinetics of the endocytic tracer FM4-64 in the roots of seedlings grown on sucrose or no-sugar medium. After 5 min, in seedlings grown on no-sugar medium, the tracer signal was mostly restricted to the plasma membrane, whereas in seedlings grown on sucrose medium, FM4-64 had started labeling endosomes (Figure 2A). After 15 min of internalization, FM4-64 had labeled the plasma membrane and endosomes in both groups of seedlings. However, labeled endosomes were more abundant when sucrose was present. Importantly, the number and size of the intracellular compartments were significantly greater in seedlings grown on sucrose medium than in those grown on no-sugar medium at each evaluated timepoint (Figure 2A). Quantitative analysis of internalized FM4-64 confirmed that FM4-64 levels in root cells were significantly higher in sucrose-grown seedlings than in seedlings grown on no-sugar medium (Figure 2B).

The first destination of endocytosed vesicles is the early endosome/trans-Golgi network (EE/TGN) (Rosquete et al., 2018). Our results suggest that sucrose induces membrane trafficking from the plasma membrane to the EE/TGN. To support this finding, we evaluated the effect of sucrose on the internalization of the auxin transporter PIN-FORMED2 (PIN2). Plasma membrane PIN2 is internalized toward the EE/TGN, where it is either recycled back to the plasma membrane or directed to the vacuole (Adamowski and Friml, 2015). PIN2 endocytosis to the vacuole is induced by darkness (Laxmi et al., 2008). We used this physiological response to monitor the endocytosis of GFP-tagged PIN2 (PIN2-GFP). To ensure that we visualized the PIN2-GFP endocytic pathway exclusively, we added cycloheximide to inhibit synthesis of new PIN2-GFP. After a 6-h dark treatment, PIN2-GFP was detected mainly at the plasma membrane and endosomes in seedlings grown in no-sugar medium (Figure 2C), but accumulated in the vacuole in seedlings grown in the presence of sucrose (Figure 2C). The intracellular signal was significantly higher in seedlings grown in sucrose medium than in those grown in no-sugar medium (Figure 2D), confirming that the presence of sucrose induces endocytosis of lipids and cargo proteins from the plasma membrane.

### Sucrose targets clathrin-mediated endocytosis dynamics

**FIGURE 3:**
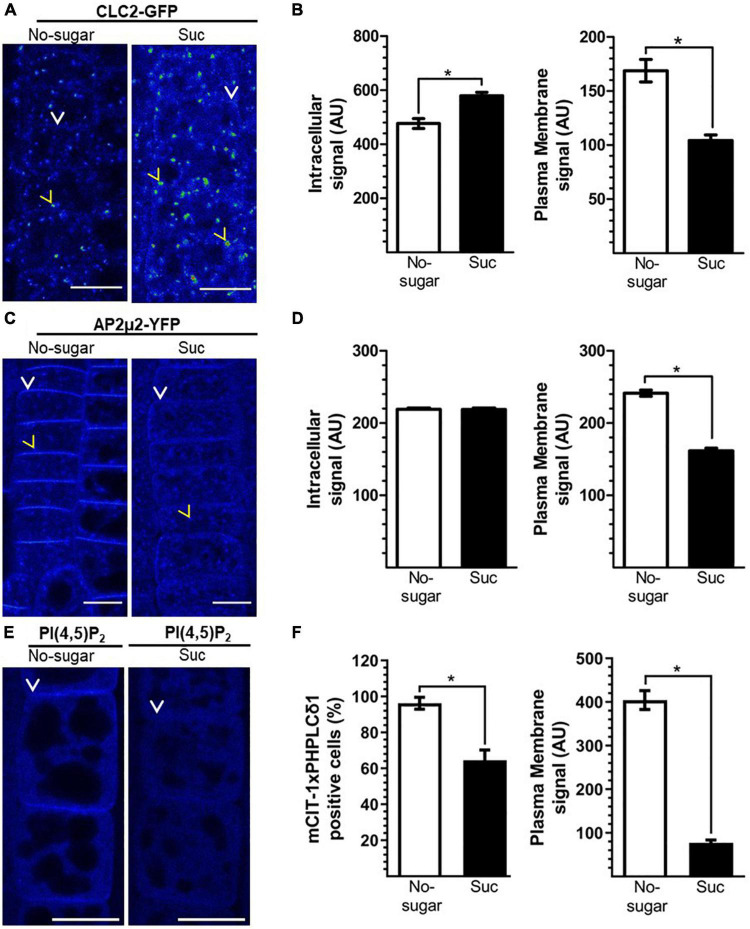
Sucrose modulates clathrin-mediated endocytosis (CME). Transgenic Arabidopsis seeds were sown on sugar-free (No-sugar) or sucrose-supplemented (60 mM, Suc) solid medium. After 7 days, seedling root epidermal cells were visualized by confocal microscopy. **(A,C)** Cellular distribution of key CME molecular players CLC2-GFP **(A)** AP2μ2-YFP **(C)** and the PI(4,5)P_2_ biosensor **(E)**. **(A,C,E)** Representative images are shown using the Rainbow RGB LUT (Fiji) of *n* = 15 seedlings in each condition. Yellow arrowheads and white arrows indicate endosomes and the plasma membrane, respectively. Bar, 10 μm. **(B,D)** Fluorescent signal at the intracellular and plasma membrane compartments was quantified in CLC2-GFP **(B)** AP2μ2-YFP **(D)** seedlings. **(F)** Fluorescent signal of the PI(4,5)P_2_ biosensor at the plasma membrane was quantified in mCIT-1xPH^PLCδ^ ^1^ seedlings. The number of analyzed cells that display plasma membrane fluorescence over the background are compared **(F)**. AU: arbitrary unit. Error bars represent standard error for three independent biological replicates (*n* = 15 seedlings). Two-tailed Student’s *t*-test; *p< 0.05.

CME is the primary endocytic mechanism in plants, fulfilling critical roles in growth and development (Chen et al., 2011; Fan et al., 2015). To assess the relationship between sucrose-mediated endocytosis and CME, we studied the subcellular distribution of core CME components in seedlings grown in the absence or presence of sucrose. Clathrin is a conserved heterohexameric complex of three clathrin heavy chains (CHCs) and three clathrin light chains (CLCs) required to configure the developing vesicle from the donor membrane (Chen et al., 2011). In mammals and yeast, CLCs play a regulatory role in the formation of the clathrin coating complex (Das et al., 2021). Therefore, we evaluated the distribution of CLATHRIN LIGHT CHAIN2 (CLC2), one of the three CLCs encoded by the Arabidopsis genome (Chen et al., 2011), by visualizing a CLC2-GFP fusion protein (Wang et al., 2013). CLC2-GFP was distributed at the plasma membrane and EE/TGN in both conditions (Figure 3A). However, the CLC2-GFP signal associated with EE/TGN was significantly higher in seedlings grown in the presence of sucrose (Figure 3B). Concomitantly, the CLC2-GFP signal at the plasma membrane was lower in seedlings grown in the presence of sucrose than in those grown without sucrose (Figure 3B), suggesting that vesicle formation at the plasma membrane occurs at a higher rate when sucrose is present. The drug brefeldin A (BFA) provokes the formation of so-called BFA bodies due to the clustering of the EE/TGN (Robinson et al., 2008). After 1 h of BFA treatment, the number and size of CLC2-GFP-labeled BFA bodies was significantly greater in seedlings grown in the presence of sucrose than in those grown without sucrose, supporting the higher level of CLC2-GFP associated with EE/TGN under sucrose conditions (Supplementary Figure 2). Importantly, the presence of sucrose did not affect the intracellular CHC protein level or distribution, or the transcript levels of *CLC* and *CHC* genes in Arabidopsis seedlings (Supplementary Figure 3).

Recognition of target proteins on the plasma membrane by adapter complexes is crucial for CME initiation. The AP2 complex recognizes conserved motifs in the cytoplasmic domain of target proteins through the AP2μ2 subunit (Chen et al., 2011; Fan et al., 2015). In addition, the AP2 complex recruits clathrin components, fulfilling a critical dual function during CME (Redlingshöfer and Brodsky, 2021). Therefore, we evaluated AP2 subcellular distribution in both growth conditions (with and without sucrose) using the AP2μ2-YFP reporter line (Bashline et al., 2013). We did not detect differences in intracellular levels of AP2μ2-YFP between the two conditions. However, AP2μ2-YFP was less abundant at the plasma membrane in seedlings grown in the presence of sucrose than in those grown without sucrose (Figures 3C,D); this was similar to the results for CLC2-GFP (Figures 3A,B). CME depends on the abundance of PI(4,5)P_2_ at the plasma membrane (Zhao et al., 2010; Li et al., 2020). Plasma membrane PI(4,5)P_2_-enriched domains are crucial for the recognition of target proteins by the AP2 complex (Rohde et al., 2002). Using the PI(4,5)P_2_ biosensor mCIT-1xPH^*PLC*δ1^ (Simon et al., 2014), we observed that plasma membrane PI(4,5)P_2_ levels were lower in seedlings grown on sucrose medium than in seedlings grown on no-sugar medium (Figures 3E,F). Indeed, the fluorescence of the biosensor at the plasma membrane dropped to undetectable levels in about of 35% of the scored cells of seedlings grown on sucrose medium (Figures 3E,F). These results are consistent with the levels of AP2μ2-YFP and CLC2-GFP under the two conditions of sucrose.

**FIGURE 4:**
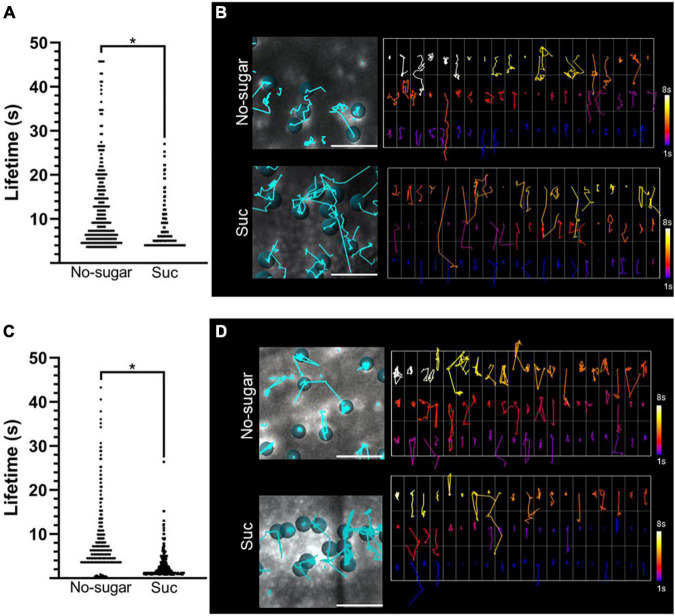
Plasma membrane residence times of CLC2-GFP and AP2μ2-YFP are reduced by sucrose. CLC2-GFP and AP2μ2-YFP transgenic Arabidopsis seeds were sown on sugar-free (No-sugar) or sucrose-supplemented (60 mM; Suc) solid medium. The dynamics of CLC2-GFP and AP2μ2-YFP at the plasma membrane were analyzed by TIRF microscopy. **(A–C)** Plasma membrane lifetimes of CLC2-GFP **(A)** and AP2μ2-YFP **(C)** foci were evaluated. Total data distribution by condition is shown (*n* ≥ 5 seedlings). Student’s *t*-test; *p< 0.05. **(B–D)** Tracking of CLC2-GFP **(B)** and AP2μ2-YFP **(D)** foci on TIRF images is shown (left panels). Cyan lines show trajectory of representative foci indicated by dots. The plasma membrane residence time for each tracked foci is shown as a code color scale (right panels). Code color scale is shown.

In summary, we showed that sucrose promotes a higher rate of endocytosis (Figure 2) and a lower abundance of CME molecular components at the plasma membrane (Figure 3). This could be explained by faster recruitment of CME components and vesicle budding. This would result in a lower plasma membrane residence time of CME components, such as CLC and AP-2 proteins. To test this hypothesis, we used total internal reflection fluorescence (TIRF) microscopy to assess the half-lives of CLC2-GFP and AP2μ2-YFP in seedlings grown in the absence or presence of sucrose. Under both conditions, CLC2-GFP was distributed in two types of foci at the plasma membrane according to their size, consistent with previous reports (Johnson and Vert, 2017; Johnson et al., 2020). Large foci with a diameter>1.5 μm were poorly mobile. Foci with a diameter of 0.5–1 μm were laterally dynamic, and after some time, their signal disappeared, showing functional endocytic foci. In contrast, AP2μ2-YFP foci were all 0.5–1 μm in diameter. The number of endocytic events in a window time was similar in the absence or presence of sucrose for both CME components (Supplementary Figure 4). We observed a significant decrease in both CLC2-GFP and AP2μ2-YFP lifetime at the plasma membrane in root epidermal cells of seedlings grown in the presence of sucrose compared to those grown in the absence of sucrose (Figures 4A,C). The half-lives average of CLC2-GFP and AP2μ2-YFP were 57,1 and 39% lower, respectively, in seedlings grown in the presence compared to the absence of sucrose (Figures 4A,C). The presence of sugar in the media promoted the increase of CME events with short half-lives compared to the no-sugar condition (Figure 4). The long lifetime events observed in seedlings grown in no-sugar media were less abundant in seedling grown in presence of sugar (white tracks, Figures 4B,D). Overall, we conclude that sucrose controls the kinetics of endocytic clathrin-coated vesicle formation and internalization in root epidermal cells.

**FIGURE 5:**
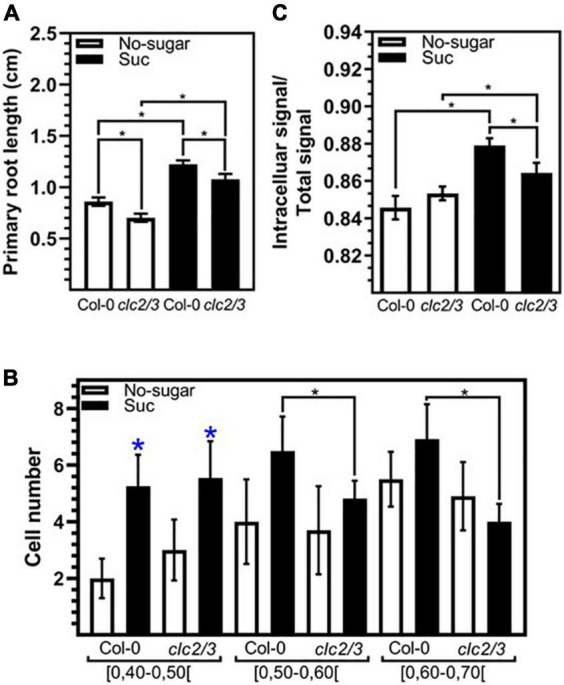
The function of *CLC2* and *CLC3* are required for sucrose induced endocytosis and cell elongation. Arabidopsis wild-type (Col-0) and *clc2-1 clc3-1* (*clc2/3*) seedlings were sown on sugar-free (No-sugar) or 60 mM sucrose-supplemented (Suc) solid medium and grown for 7 days. **(A)** Primary root length of seedlings was measured in *n* ≥ 20 seedlings per replicate. **(B)** Cell height and width were measured for epidermal cells on the meristematic zone (ratio height/width ≤ 1) of the seedling primary root. The number of cells that display a range of a cell elongation index is shown. *n* ≥ 15 seedlings were scored in either condition. **(C)** Endocytosis of FM4-64 was evaluated by confocal imaging of epidermal cells after 15 min of FM4-64 plasma membrane labeling. About 15 seedlings were evaluated for either condition in experimental triplicates. FM4-64 was quantified at the intracellular and whole-cell. Results are expressed as the ratio between both values. **(A–C)** Error bars represent standard error for three independent biological replicates. **(A,B)** Student’s *t*-test; *p< 0.001. **(C)** Two-tailed-Student’s *t*-test; *p< 0.05. Blue asterisks indicate statistical differences between no-sugar and sucrose condition for each line.

Simultaneous loss of function of *CLC2* and *CLATHRIN LIGHT CHAIN3 (CLC3)* results in inhibition of both root growth and plasma membrane internalization (Wang et al., 2013). To evaluate the requirement of CME for root cell elongation modulated by sucrose, we tested the response of the double mutant *clc2-1 clc3-1* (Wang et al., 2013) to this sugar. *clc2-1 clc3-1* seedlings grown in the presence of 60 mM sucrose developed longer roots than those grown in the absence of sucrose (Figure 5A). This indicates that the double mutant was still sensitive to sucrose. However, the primary root of *clc2-1 clc3-1* was shorter than Col-0 in both sucrose and non-sugar condition (Figure 5A). Then, we evaluated the number of meristematic cells of each category of cell elongation index similarly to the analysis performed for Col-0 in Figure 1E. Double mutant seedlings grown in sucrose medium had a significantly less cells with an elongation index of 0.50–0.70 than Col-0 seedlings grown in the same condition (Figure 5B). In contrast, no differences between Col-0 and *clc2-1 clc3-1* were found in shorter cells (cell elongation index<0.5) (Figure 5B). The lower number of long meristematic cells is consistent with a shorter root in the double mutant *clc2-1 clc3-1*. These results point out that the function of *CLC2* and *CLC3* is important for the promotion of root cell elongation. The effect of the loss of function of *CLC2* and *CLC3* on cell elongation could be explained by the defect on CME. Indeed, *clc2-1 clc3-1* seedlings grown in the presence of sucrose displayed a lower endocytosis rate compared to Col-0 seedlings grown in the same condition (Figure 5C). The double mutant was still able to increase the endocytosis in response to sucrose (Figure 5C) most likely by a compensatory role of the CLATHRIN LIGHT CHAIN1 (CLC1). Overall, the results indicate that a functional CME pathway is required for cell elongation in response to sucrose.

### Sucrose modulation of clathrin-mediated endocytosis involves a physiological response

**FIGURE 6:**
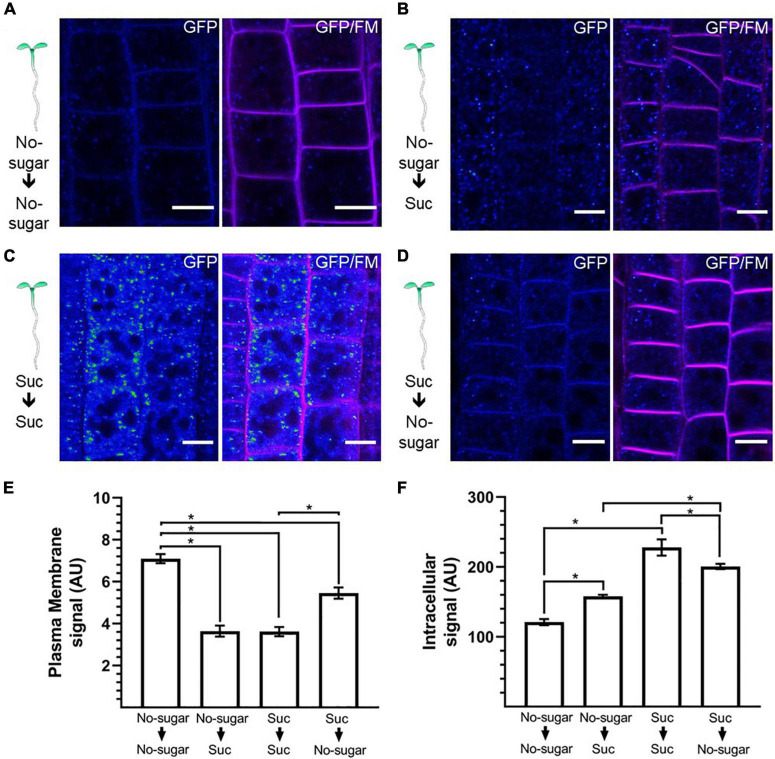
Endocytosis is dynamically and reversibly modulated by sucrose. Arabidopsis transgenic CLC2-GFP plants were grown for 7 days on sugar-free (0 mM; No sugar) **(A,B)** or sucrose-supplemented (60 mM; Suc) **(C,D)** solid medium. Seedlings were transferred to sugar-free **(A–D)** or sucrose-containing medium **(B,C)** for 24 h. The cellular distribution of CLC2-GFP was evaluated in root epidermal cells. The plasma membrane was labeled with FM4-64. Representative confocal images of *n* = 15 seedlings under each condition are shown. The GFP signal is shown as Rainbow RGB LUT (Fiji). Bar, 10 μm. **(E,F)** The fluorescent signal at the plasma membrane **(E)** and the intracellular compartments **(F)** was quantified in CLC2-GFP seedlings. AU: arbitrary unit. Error bars represent standard error for three independent biological replicates (*n* = 15 seedlings). Two-tailed Student’s *t*-test; *p< 0.05.

Abiotic conditions, such as reductions in light incidence during the daily cycle, restrict photosynthesis, and sugar levels in sink organs fluctuate accordingly (Rosa et al., 2009; Vialet-Chabrand et al., 2017). We showed that sucrose modulates CME in seedlings germinated and grown in the absence or presence of sucrose. Next, we tested whether sucrose-modulated CME could be induced and reversed in response to fluctuations in sucrose that occur naturally during a plant’s lifespan. We followed the distribution of CLC2-GFP as a reporter for CME. Seedlings were first grown on either no-sugar or sucrose medium and then transferred to fresh medium for 24 h. When seedlings were transferred from no-sugar medium to sucrose medium, the plasma membrane-associated CLC2-GFP signal was weaker than when seedlings were transferred from no-sugar medium to no-sugar medium, confirming that the effect of sucrose is an inducible response (Figures 6A,B,E). Moreover, when seedlings were transferred from no-sugar medium to sucrose medium, most of the signal was observed in the EE/TGN (Figures 6B,F). When seedlings grown on sucrose medium were transferred to no-sugar medium, seedlings recovered plasma membrane localization of CLC2-GFP, indicating that modulation of CME is reversible (Figures 5D,E). Therefore, CME modulation could be a response to temporal sucrose fluctuations, controlling root growth depending on energy availability and plant performance.

**FIGURE 7:**
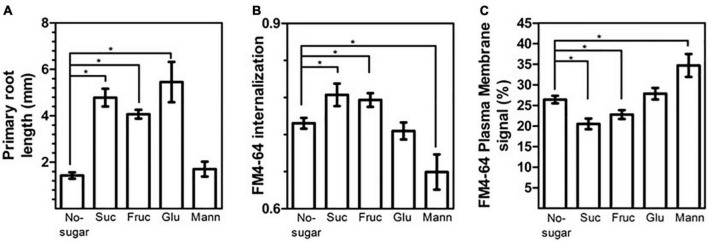
The sucrose monosaccharide fructose mimics the effect of sucrose on cell elongation and endocytosis. Wild-type Arabidopsis (Col-0) seeds were sown on sugar-free solid medium (No-sugar) and solid medium supplemented with 60 mM sucrose (Suc), fructose (Fruc), glucose (Glu) or mannitol (Mann). **(A)** Primary root length of seedlings was scored after 7 days from three independent biological replicates of *n* ≥ 20 seedlings for each condition. **(B,C)** Endocytosis was evaluated in root epidermal cells from 15 seedlings from each condition in three independent replicates. The plasma membrane, endosome, and whole-cell FM4-64 signal was quantified manually from confocal images after 30 min of FM4-64 internalization. **(B)** FM4-64 internalization was calculated as the intracellular signal relative to the whole-cell signal. **(C)** The plasma membrane signal relative to the whole-cell signal is shown as a percentage. **(A–C)** Error bars represent standard error. **(A)** Student’s *t*-test; *p< 0.05. **(B,C)** Two-tailed Student’s *t*-test; *p< 0.05.

Sucrose can be broken down into fructose and glucose by cell-wall-localized invertases before intracellular transport (revised at Pego and Smeekens, 2000; Roitsch and González, 2004; Ruan et al., 2010). Therefore, we explored whether sucrose metabolites could also modulate root growth and CME. We determined the response of primary roots and FM4-64 internalization in wild-type seedlings grown on medium supplemented with either fructose or glucose at the same concentration tested for sucrose (60 mM). Both sugars promoted primary root growth (Figure 7A). After 7 d of growth, roots of seedlings grown with fructose, sucrose, or glucose were 2.8, 3.3, and 3.8 times longer, respectively, than those of seedlings grown in no-sugar medium (Figure 7A).

Seedlings grown in fructose medium displayed greater FM4-64 internalization than seedlings grown in no-sugar medium (Figure 7B). Consistent with this result, the level of FM4-64 at the plasma membrane was lower in seedlings grown in fructose medium (Figure 7C). The effect of fructose was similar to that of sucrose, suggesting a similar mechanism for inducing endocytosis (Figures 7B,C). No significant differences in FM4-64 internalization or FM4-64 abundance at the plasma membrane were observed between seedlings grown in glucose medium and in no-sugar medium, indicating that glucose signaling is not involved in sucrose modulation of CME (Figures 7B,C). Moreover, mannitol, which is an unrelated sugar, did not induce either primary root elongation or plasma membrane internalization when it was present in the same concentration (Figure 7), indicating that the effect of fructose and sucrose is specific. Indeed, the presence of mannitol in the growth medium inhibited FM4-64 internalization (Figures 7B,C). This is consistent with the perturbation of endocytic trafficking due to mannitol-induced osmotic stress (Zwiewka et al., 2015). Therefore, the fact that sucrose and fructose trigger equivalent physiological and cellular effects is not due to osmotic stress.

Overall, these data support the role of sucrose in root growth through CME modulation, indicating that sucrose is a mobile signal. Sucrose levels likely regulate communication between source and sink organs, resulting in a physiological response to the energy status and/or metabolism of a plant.

## Discussion

Endocytosis regulates different physiological processes in plants through protein internalization and turnover from the plasma membrane. Here, we show that the presence of sucrose induces cell elongation and endocytic trafficking in the epidermal cells of Arabidopsis roots. In particular, sucrose targets CME by modulating the kinetics of vesicle formation and internalization. This effect is inducible and reversible, suggesting a physiological response to fluctuations in sucrose availability. The effect is specific to sucrose and its monosaccharide fructose, indicating that this constitutes a different mechanism from glucose signaling. CME modulation by sucrose suggests the existence of a sucrose-sensing mechanism at the plasma membrane and/or apoplast. This mechanism would drive a cellular response, resulting in cell elongation through the endocytic pathway.

## Sucrose as a regulator of endocytic trafficking

Our results showed that exogenous sucrose promotes membrane and protein endocytosis from the plasma membrane to the EE/TGN (Figure 2), similar to the fluid-phase endocytosis induction observed in cultured cells of *Acer japonicus* (Etxeberria et al., 2005). More specifically, sucrose targets key components of CME for endocytic vesicle formation (Figure 3). The lower levels and shorter plasma membrane residence times of AP2 and CLC in the presence of sucrose are indicative of an increase in vesicle formation and internalization toward the EE/TGN. The fact that AP2 vesicles are affected suggests that sucrose-modulated CME drives protein internalization; however, the specificity of this process and the identity of cargo proteins remain unclear. Recruitment of AP2 at the endocytic pit requires PI(4,5)P_2_-enriched plasma membrane domains, which may be important in the regulation of CME (Redlingshöfer and Brodsky, 2021). Our results show a lower abundance of PI(4,5)P_2_ associated with a higher rate of vesicle internalization (Figures 3, 5) in the presence of sucrose, suggesting that the metabolism of phosphoinositides is a primary target for CME modulation by this sugar.

The effect of sugar on CME was specific to sucrose and fructose. The monosaccharides glucose and mannitol had no effect on plasma membrane internalization (Figure 6). Moreover, mannitol, which is a non-metabolized sugar, inhibited endocytosis, suggesting that the trafficking modulation by sucrose is independent of an osmotic stress effect. Consistent with our results, sucrose, but not glucose, induces endocytosis in non-photosynthetic cultured cells (Etxeberria et al., 2005). In our study with Arabidopsis seedlings, fructose promoted endocytosis, similar to sucrose (Figure 6). However, fructose had no effect on cultured cells (Etxeberria et al., 2005). This result reveals that promotion of CME is due to sugar availability rather than the breakdown of sucrose. Indeed, equal molarity of fructose or sucrose promoted different levels of CME induction. The fact that fructose supplementation induces lower levels of CME indicates that these sugars are sensed or mediated by a different molecular mechanism.

Sucrose is an early growth signal in seedling roots, where cell division and elongation must occur in coordination (Kircher and Schopfer, 2012). An intricate network of hormones, including auxin, cytokinins, gibberellin, and brassinosteroids, play essential roles in root cell division and elongation (Takatsuka and Umeda, 2014; Oh et al., 2020). In particular, endocytosis has a pivotal role in the modes of action of auxin and brassinosteroids, regulating the levels of hormone transport and signal perception, respectively (Kitakura et al., 2011; Irani et al., 2012). In our study, exogenous sucrose promoted cell elongation and, consequently, root growth (Figure 1). Cell elongation could not be explained by a higher rate of internalization of PIN proteins or BRI1. Indeed, enhancing the endocytic trafficking of such proteins results in inhibition of primary root elongation (Pérez-Henríquez et al., 2012; Yu et al., 2020). Sucrose-induced PIN2-GFP internalization promotes root growth (Figures 1, 2); therefore, hormones as a main target of sucrose-modulated CME is unlikely.

Physiological conditions modulate endocytosis of different pools of cargo proteins. For instance, salt stress induces internalization of the aquaporin PIP2;1, limiting water permeability and, consequently, cell elongation (Ueda et al., 2016). Darkness and root bending induce internalization of auxin facilitators (Laxmi et al., 2008; Rakusová et al., 2011). Following a wounding stimulus, CME of the ligand-receptor complex PLANT ELICITOR PEPTIDE (PEP) 1/PEP RECEPTOR 1 is induced to turn on cell signaling (Ortiz-Morea et al., 2016; Jing et al., 2019). The specific pool of proteins internalized under sucrose/fructose availability would determine the cell response and/or trigger a signaling pathway. This would not be due to cellular energy status, since supplementation of glucose does not provoke the same effect on CME.

### Potential signaling mechanism mediated by a sucrose-modulated clathrin-mediated endocytosis pathway

Sucrose is mobilized from photosynthetically active tissues to sink organs. Environmental factors impact photosynthesis, determining the sucrose levels destined for the sink organ. Our experimental conditions, in which we added exogenous sucrose, mimic the effect of a fully functional and actively photosynthesizing aerial tissue. Such conditions have been widely used for *in vitro* seedling growth, as well as for the characterization of responses and molecular pathways modulated by sucrose (Yoon et al., 2021). We observed sucrose modulation of CME in seedlings germinated and grown in the presence of sucrose, and found that CME could be induced within 24 h in seedlings grown in the absence of sucrose by transferring them to sucrose medium (Figure 5). More importantly, this CME modulation is a reversible process, indicating an on/off cellular mechanism. The role of sucrose as a long-distance signal for root growth by photosynthesis has been described (Kircher and Schopfer, 2012); however, the cellular mechanism is still elusive. In this context, CME could have a pivotal role in mobilizing molecular components within cell compartments as part of a signaling mechanism.

Sucrose triggers global transcriptional changes, and effects on molecular players such as transcription factors have been described (Jeong et al., 2010; Shin et al., 2013). Sucrose regulates its own uptake from the extracellular space, determining the extent of the transcriptional response (Etxeberria et al., 2005; Jeong et al., 2010; Shin et al., 2013). This response is conditioned by a transient increase in cytoplasmic calcium within a few minutes of the sucrose stimulus (Shin et al., 2013). In addition, sucrose supplementation promotes autophosphorylation of the plasma membrane receptor SUCROSE INDUCED RECEPTOR KINASE 1 (SIRK1), triggering its interaction with and phosphorylation of the PIP2F aquaporin during cell elongation (Niittylä et al., 2007; Wu et al., 2013). This also happens with the sucrose transporter SWEET11 in Arabidopsis root cells, suggesting that the regulation of sucrose transport is mediated by SIRK (Wu et al., 2013). Notably, SIRK1 kinase is endocytosed after phosphorylation and directed to the early endosome in a pathway modulated by sucrose levels (Wu et al., 2013). Most likely, sucrose-induced CME is responsible for SIRK1 endocytosis since the interaction of SIRK1 with CHC1, CHC2, and CLC2 has been reported. The SIRK1-clathrin interaction is constitutive and strengthened by sucrose stimuli (Wu et al., 2013). Endocytosis of SIRK1 could be involved in endosome signaling for processes such as cell elongation and root growth. Signaling from endosomes has been widely reported in mammals (Villaseñor et al., 2016), but there have been few examples in plants (Sharfman et al., 2011; Mbengue et al., 2016).

The transcriptional response modulated by sucrose requires the plasma membrane sucrose transporter SUT1 (Shin et al., 2013). Two different pools of SUT1 are present at the plasma membrane: dimers of SUT1 in lipid raft domains and monomers in a sterol-independent membrane domain (Liesche et al., 2010). The level of SUT1 at the plasma membrane is regulated by the endocytosis and recycling of SUT1 dimers in a lipid-raft-dependent pathway (Krügel et al., 2008). In addition, the monomeric form of SUT1 could be subject to CME-dependent trafficking as observed for aquaporins such as PIP2;1, with a dual lipid distribution at the plasma membrane (Li et al., 2011). This suggests a sucrose signaling arm involving SUT1 regulation by EMC.

## Data availability statement

The original contributions presented in this study are included in the article/Supplementary material, further inquiries can be directed to the corresponding author/s.

## Author contributions

CO-N: conception and design of the work, data analysis and interpretation, drafting, and critical revision of the article. JT: data analysis and interpretation. LN: conception and design of the work, data interpretation, and writing of the article. All authors contributed to the article and approved the submitted version.

## Footnotes

1
http://fiji.sc
