# Supplementary material for: Sucrose targets clathrin-mediated endocytosis kinetics supporting cell elongation in Arabidopsis thaliana
Source: Front Plant Sci. 2022 Oct 18;13:987191. doi: 10.3389/fpls.2022.987191 (PMC9623095; doi:10.3389/fpls.2022.987191)
Supplement: Supplementary file 1 [file Presentation_1.pdf]

Supplementary Material

Supplementary methods

Immunofluorescence: Whole-mount root seedling immunodetection was performed as described by Sauer et al. (2006). Anti-CHC (α-CHC) antibody (Dhonukshe et al., 2007) was used at a 1:400 dilution, and anti-rabbit IgG Cy3 conjugated antibody (Sigma-Aldrich) was used as a secondary antibody at a 1:600 dilution. Seedlings were observed by confocal microscopy.

Transcript level quantification: Total RNA was isolated using a RNeasy Plant Mini Kit (QIAGEN) according to the manufacturer's recommendations. cDNA was generated using Impron (Promega) with 1 µg of purified RNA. qPCR was carried out using Brilliant III Ultra-Fast qPCR Master Mix (Agilent Technologies) in a Stratagene MX3000P (Agilent Technologies). Primer designs were evaluated using the Oligoanalyzer tool from IDT (http://eu.idtdna.com/calc/analyzer). Primers are listed in Supplementary Table 1. *ACTIN2* (AT3G18780) was used as a housekeeping gene. Transcript level was determined by the ΔΔCt method (Pfaffl, 2001).

Supplementary references

Dhonukshe, P., Aniento, F., Hwang, I., Robinson, D.G., Mravec, J., Stierhof, Y.D., et al. (2007) Clathrin-mediated constitutive endocytosis of PIN auxin efflux carriers in Arabidopsis. Curr. Biol. 17, 520-527. doi: 10.1016/j.cub.2007.01.052

Sauer, M., Paciorek, T., Benková, E., and Friml, J. (2006) Immunocytochemical techniques for whole-mount in situ protein localization in plants. Nat. Protoc. 1, 98-103. doi: 10.1038/nprot.2006.15

Pfaffl, M.W. (2001). A new mathematical model for relative quantification in real-time RT-PCR. Nucleic Acids Research 29: e45.

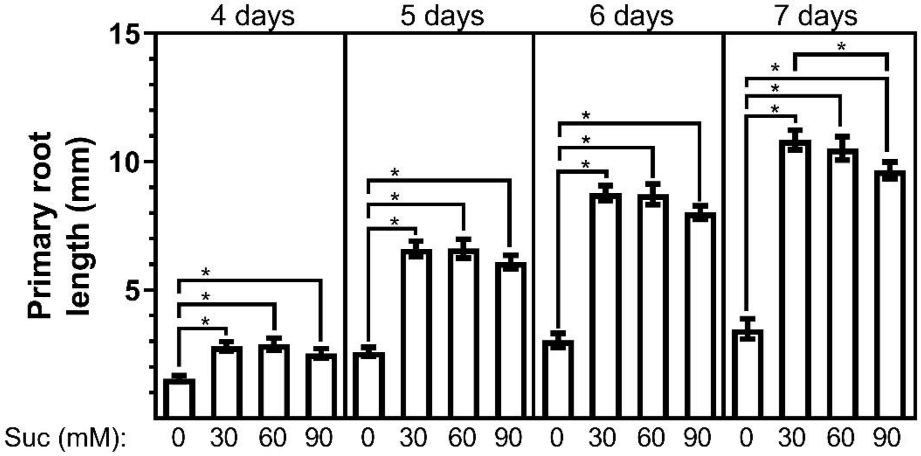

Supplementary Figure 1. **Elongation of root epidermal cells is promoted by sucrose**. Wild-type Arabidopsis (Col-0) seeds were sown on solid medium without sugar (0) or supplemented with 30, 60 or 90 mM sucrose and grown for several days as indicated. Primary root length of seedlings was measured in $n \geq 20$ seedlings per replicate. Error bars represent standard error. Student's *t*-test; *, $p < 0.05$.

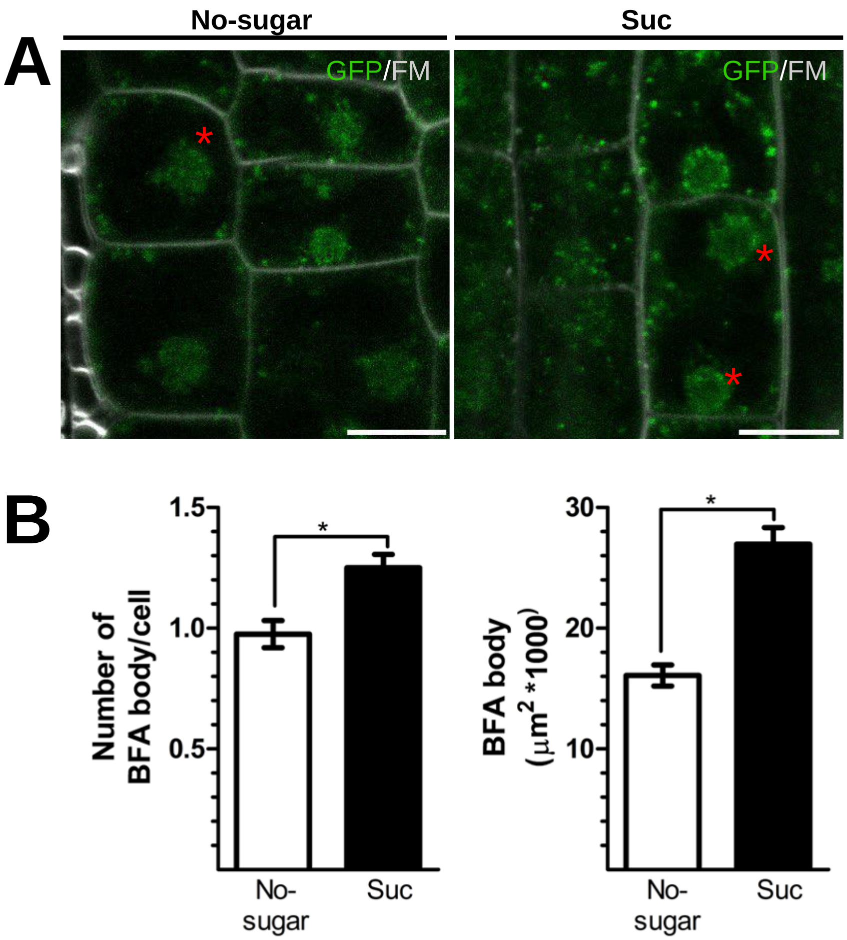

Supplementary Figure 2. Sucrose promotes clathrin-mediated endocytosis in root epidermal cells. Arabidopsis CLC2-GFP seeds were sown on sugar-free (No-Sugar) or 60 mM sucrose-supplemented (Suc) solid medium and grown for 7 d. Seedlings were incubated in 50 µM cycloheximide for 30 min. Brefeldin A was added (50 µM) to block membrane recycling and endosome clustering. Seedlings were incubated for an additional 60 min. **(A)** CLC2-GFP cell distribution was evaluated by confocal microscopy. FM4-64 was used for plasma membrane visualization. Red asterisks indicate the CLC2-GFP-labeled BFA body. Representative confocal images from three independent biological replicates are shown ($n \geq 20$ seedlings for each condition). Bar, 10 µm. **(B)** The number and the area of the BFA bodies was scored using FIJI. Error bars represent standard error. Two-tailed Student's *t*-test; *, $p < 0.05$.

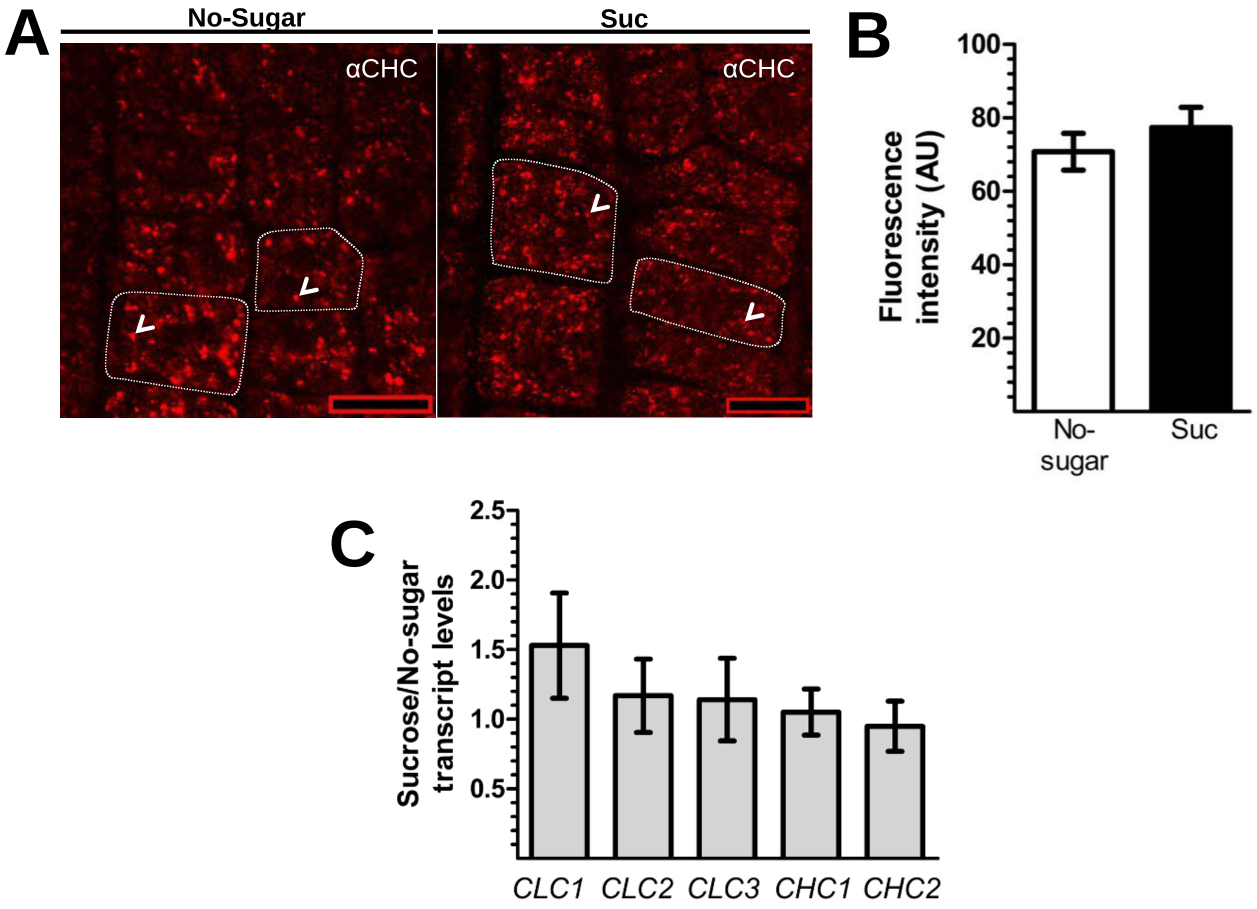

Supplementary Figure 3. Sucrose does not affect the level and distribution of clathrin proteins. Wild-type (Col-0) and *α*CHC2-GFP seeds were sown on sugar-free (No-sugar) or 60 mM sucrose-supplemented (Suc) solid medium and grown for 7 d. **(A)** The distribution of *α*CHC2-GFP was evaluated by immunofluorescence using specific antibodies and confocal microscopy. Representative confocal images from three independent biological replicates are shown ($n \geq 20$ seedlings for each condition). The cell limit was outlined manually for better visualization (dotted line). Bar, 10 µm. **(B)** Fluorescence from the images in A was quantified using Fiji. Error bars represent standard error. No statistical differences were found after Two-tailed Student's *t*-test **(C)** Transcript level of *CLC* and *CHC* under both conditions was determined by RT-qPCR. Results are expressed as the ratio between the levels in the sucrose and no-sugar conditions. RNA was isolated from three independent biological replicates. RT-qPCR analysis was performed in triplicates. **(B, C)** Error bars represent standard error. No statistical differences were found after one-way ANOVA with post-hoc Tukey test.

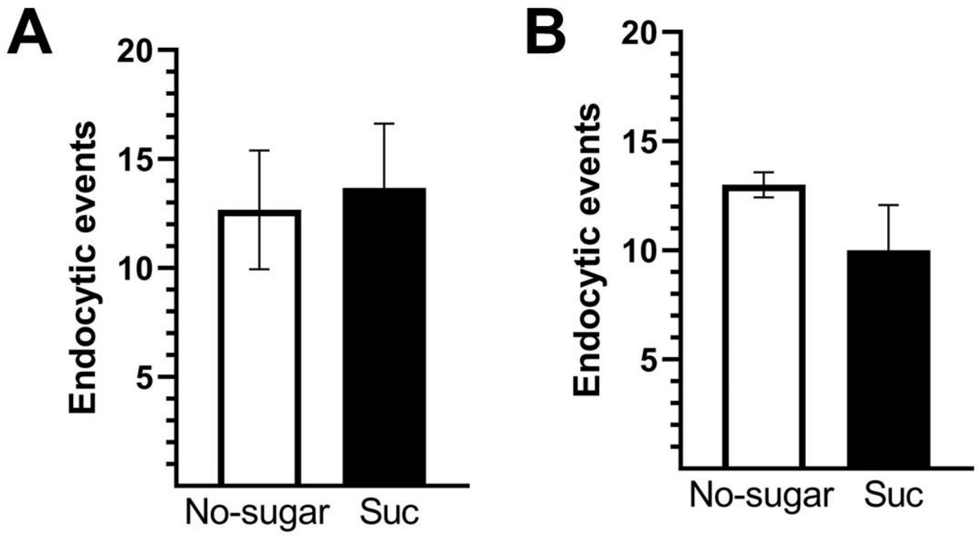

Supplementary Figure 4. Sucrose does not affect the number of CME events in a period. CLC2-GFP and AP2μ2-YFP transgenic Arabidopsis seeds were sown on sugar-free (No-sugar) and sucrose-supplemented (60 mM; Suc) solid medium. Plasma membrane protein residence was analyzed by TIRF microscopy. The number of endocytic events of CLC2-GFP **(A)** and AP2μ2-YFP **(B)** were calculated in a window of 40 seconds from tracking analysis. No statistical differences between no-sugar and sucrose condition were found after two-tailed Student's *t*-test; *, $p < 0.05$.

Supplementary Table 1. List of primers used in this work.

Gene	TAIR ID		Primer sequence
CLC1	AT2G20760	Forward	TCACCTGCCAAAGACGCAAGTGTTGA
		Reverse	CTCCGCCTTGGTTCCCTCGGCCTCSGT
CLC2	AT2G40060	Forward	AAGAATGTTCCCGAAACCAAG
		Reverse	TCACACAGATGTAAGCAACGA
CLC3	AT3G51890	Forward	CCACTGATCTGTCTCGTATGCGT
		Reverse	CACTCACACTCACATTCGGGTCAG
CHC1	AT3G11130	Forward	ACAAAAAGAGGTGAAGGCCAAAGA
		Reverse	TTCCTGACATTCCTGGCATTCCA
CHC2	AT3G08530	Forward	ACAAAAAGAGGTGAAGGCCAAAGA
		Reverse	TGGCATGGGAGGCATTCCTGGCATACCG
ACTINA2	AT3G18780	Forward	GCTTCTCCATTTGTTTGTTTCAT
		Reverse	GGAATAAAGAGGCATCAATTCGA
UBIQUITINA10	AT4G05320	Forward	ACCAGCAGCGTCTCATCTT
		Reverse	GCATAACAGAGACGAGATTTAGAA
PP2A	AT1G13320	Forward	GATACTTTGTCGTGTGTTGTCTT
		Reverse	CAAATACGCCCAACGAACAA
